# State of hormonal balance in adolescent girls with menstrual function disorders associated with obesity

**DOI:** 10.25122/jml-2021-0312

**Published:** 2021

**Authors:** Alla Volodymyrivna Borshuliak, Oksana Anatoliivna Andriiets, Oksana Valerianivna Bakun, Anatolii Volodymyrovich Andriiets, Volodymyr Vasyliovich Andriiets, Michael Ivanovicha Sheremet, Valentin Nicolae Varlas

**Affiliations:** 1. Kamyanets-Podilsky Medical Professional College, Kamyanets-Podilsky, Ukraine; 2. Department of Obstetrics and Gynecology, Bukovinian State Medical University, Chernivtsi, Ukraine; 3. Surgery department No.1, Bukovinian State Medical University, Chernivtsi, Ukraine; 4. Department of Obstetrics and Gynecology, Carol Davila University of Medicine and Pharmacy, Bucharest, Romania

**Keywords:** obesity, hormone levels, menstrual function, hyperandrogenism, MFI – menstrual function impairment, BMI – body mass index, LH – luteinizing hormone, FSH – follicle-stimulating hormone, PCOS – syndrome of polycystic ovaries

## Abstract

Investigation of the mechanisms promoting the development of menstrual function disorders associated with obesity in adolescent girls is one of the most important issues of modern medicine. This study included 110 patients. 79 patients aged 12–18 with menstrual disorders associated with obesity were divided into two groups: group 1: 46 patients with apparent signs of hyperandrogenism, group 2: 33 patients without clinical manifestations of hyperandrogenism. The control group included 31 girls of the same age with a regular menstrual cycle. The complex of hormone examination of adolescent girls included determination of serum content: gonadotropic hormones (luteinizing hormone (LH) and follicle-stimulating hormone (FSH), prolactin (PRL), estradiol (E2), testosterone (T), progesterone (PR), dehydroepiandrosterone sulfate; insulin (In) – radioisotope method on a gamma counter “Narcotest” (reagents “IMMUNOTECH”, Czech Republic). We identified hyperleptinemia and leptin resistance in patients with menstrual function impairment associated with obesity. In group I, the adiponectin level exceeded the values of the control group (p<0.05). The results revealed a decrease in A/L in group I – 5.4 times compared with patients in group II (p<0.05) and 4.3 times – compared with the control group (p<0.05). The results revealed a decrease in A/L among girls in the group I with MFI associated with obesity – 4.3 times - compared with girls in the control group (p<0,05).

## Introduction

Obesity is an important part of reproductive endocrinology, leading to various menstrual disorders since the onset of menarche. Studies show that obesity affects the age of the onset of menarche, the formation of menstrual function, and the cyclic activity of the ovaries. The progressive increase in the number of obese patients, especially in females, accompanied by various reproductive disorders, determines the relevance of studying this problem.

Despite some common features of changes in hormone metabolism in obese patients, the nuances of this pathological process depend on gender and the patient’s age. In most cases, in men who have not been diagnosed with a pituitary disorder, obesity is characterized by low levels of circulating testosterone and slightly reduced levels of luteinizing hormone (LH) and follicle-stimulating hormone (FSH) [1]. Decreased SGH levels in obese male patients correlate with increased body mass index (BMI) and insulin resistance in patients with metabolic syndrome and type 2 diabetes [2]. Excess adipose tissue in obese men increases the production of estrogen and leptin, which adversely affect the amplitude and frequency of secretion of gonadotropin-releasing factor and gonadotropic hormones (LH and FSH). In addition, in obese patients, there is a decrease of adiponectin (the content of which is inversely correlated with insulin resistance), which is also involved in regulating the secretion of gonadotropic hormones [3]. Reduction of gonadotropin stimulation of Leydig cells leads to inhibition of testosterone production. Even with normal Leydig cell function, plasma testosterone levels in obese men are lower than normal because the majority of the androgens are deposited in adipose tissue due to excessive levels of endogenous leptin, a marker of leptin receptors resistance to their ligand [4], which is a characteristic of the metabolic syndrome.

In obese women, along with a dysfunction of the metabolism of sex hormones, there is a disorder of the hypothalamic-pituitary-ovarian axis, which can cause pathological conditions such as ovulatory dysfunction and hormone-dependent malignancies (including estrogen-dependent breast cancer and endometrial cancer), and polycystic ovary syndrome. To characterize the hormonal status of obese patients, menopausal status should be considered in women, as age-related changes in the level of the hormones are critical. In young and middle-aged obese women with normal menstrual function, the total level of androgens in the blood, including testosterone, does not increase; moreover, it may be slightly reduced. On the other hand, the level of free testosterone increases significantly and can reach abnormal levels in women (especially in the abdominal-visceral type of obesity). This hormone level in blood is caused by the decrease of SGZG concentration and the increase of BMI. Adipose tissue can deposit steroids due to their high solubility in lipids, so the total amount of sex hormones in the body, including androgens, is much higher in obese patients than in people with normal weight. In addition, the conversion of androstenedione to estrone and testosterone by aromatase and 17-beta-hydroxy-steroid dehydrogenase, respectively, is active in adipose tissue, which is partly associated with conditions of functional hyperestrogenism and hyperandrogenism in obese patients [5].

Adipose tissue is involved in the metabolism of sex steroids and independently produces a number of adipocytokines and hormones, such as leptin, adiponectin, TNF-α, IL-6, and their soluble receptors [6]. BMI is a predictor of changes in leptin levels [7]. In this regard, Maffei *et al.* [8] suggested using the Leptin/BMI index (L/BMI) as an indicator of leptin resistance.

In the pathogenesis of the metabolic syndrome, along with the development of hyperinsulinemia and insulin resistance (IR), a significant role belongs to the imbalance of adipocytokines, one of which is adiponectin. Moreover, hypoadiponectinemia is accompanied by IR. Thus, determining serum adiponectin levels in adolescents with associated obesity can improve the diagnosis of IR, clarify the course of ovarian dysfunction, optimize treatment tactics, and the prognosis of menstrual recovery.

Despite the practical absence of changes in the concentration of total androgens in the bloodstream, the rate of their production and excretion changes significantly. In particular, the synthesis of testosterone, androstenedione, and other androgens in the ovaries and adrenal glands is abnormally increased, and the process of their hepatic conjugation and excretion is accelerated. An increase in the clearance of these compounds correlates with a decrease in SGZG level and androgens, which are free for metabolism. Although the effects of hyper-production and accelerated excretion of these hormones are mutually compensated, the above processes still cause the abdominal type of fat deposition in women. Also, an increase in androgen production in obese women correlates with decreased body tissue sensitivity to insulin [9].

## Material and Methods

We conducted a comprehensive clinical and laboratory examination of 79 patients aged 12–18 with menstrual disorders associated with obesity with complaints of menstrual dysfunction, delayed menstruation for up to 5 months, and 31 girls of the same age with regular menstrual cycle (reg. MC), which formed a comparison group.

### Inclusion criteria:

• Girls aged 12 to 18 years;
• Presence of menstrual disorders in adolescent girls in the presence of excess body weight (various degrees of obesity);
• No exacerbations of chronic inflammatory extragenital diseases, if any, for the period of inclusion in the study;
• Absence of other gynecological pathology;
• The voluntary consent of parents for the participation of minors in the study.

### Exclusion criteria:

• Age of patients – younger than 12 years, older than 18 years;
• Presence of exacerbations of chronic extragenital diseases;
• Presence of other gynecological pathology at the time of examination;
• History of gynecological surgical interventions;
• Presence of menstrual disorders caused by another concomitant inflammatory, including infectious, anatomical anomalies of the pelvic organs, genitals;
• People with mental disorders.

The complex of hormone examination of adolescent girls included determination of serum content: gonadotropic hormones (luteinizing hormone (LH) and follicle-stimulating hormone (FSH), prolactin (PRL), estradiol (E2), testosterone (T), progesterone (PR), dehydroepiandrosterone sulfate (performed with Best Diagnostics reagents, Ukraine), and insulin (In) – radioisotope method on a gamma counter “Narcotest” (reagents “ IMMUNOTECH”, Czech Republic).

The HOMA insulin resistance index was calculated by the formula 2.2: HOMA=(G0 × Ins0)/22.5, (2.2) where G0 is the fasting plasma glucose level, mmol/l; Ins0 – fasting serum insulin, μOD/ml.

The presence of IR in patients was diagnosed with a HOMA level higher than 3.5 conventional units [10]. Blood sampling for hormone testing in adolescent girls in the control group was performed on days 5–7 of the menstrual cycle. Blood sampling was performed in the morning (at 8.00) on an empty stomach from the elbow vein.

In order to develop differentiated treatment regimens for adolescent girls with menstrual irregularities associated with obesity, we developed an algorithm that allows the prediction of menstrual irregularities/recovery and chooses the optimal tactics for these patients. To create it, we used a dichotomous classification model built using the CART decision tree (Classification and Regression Tree). To construct the classifier, we used A/L – a parameter consisting of significant differences between the main group and the control group, which allowed us to determine which class (I – MFI IR; II – without menstrual irregularities and IR) belongs to the patient. It is suggested that counteracting the normal activity of granulosa cells in overweight individuals may lead to an increase in leptin levels above the “critical” level. The participation of adiponectin in the pathogenesis of hyperandrogenic ovarian dysfunction is proved by the data below. Thus, the level of the A/L ratio can be used to assess the metabolic status and predict its impact on the functional state of the ovaries in adolescents.

Statistical data processing was performed using a personal computer IBM RT/AT and application packages Microsoft Excel, Statgraphics Plus 3.0, SPSS Statistics 17.0, with the definition of the main statistical indicators of the series (M, m, Me, SD). The probability level of all digital indicators (P) was determined using the Student parametric criterion t. A nonparametric Fisher (φ) and Wilcoxon-Mann-Whitney (u) angular transformation criterion was used to estimate the differences in the ratios of the quantities [11]. To determine the probability of occurrence of the event, the odds ratio (VS) was used to determine the 95% confidence interval [12].

## Results

The level of gonadotropic hormones (LH and FSH) in the blood studied in 79 girls with MFI was associated with obesity and 31 of their peers with regular MC. As a result of this study fragment, we found hyperleptinemia and leptin resistance in patients with MFI associated with obesity. In the control group, leptin and L/BMI did not exceed the standards (Table 1). Hyperleptinemia is accompanied by hyperinsulinemia and IR. Therefore, hyperleptinemia and leptin in group I can be associated with hyperinsulinemia and IR, as evidenced by the established correlations between leptin and HOMA-IR (ρ=0.65), the ratio of L/BMI and Caro index (ρ=0, 8) (p<0.05).

**Table 1.** Indicators of adipokines and insulin resistance.

**Indexes**	**Main group n=79**	**Control group n=31**
**Leptin (ng/ml)**	48.4 (77.3; 39)	16.63 (27.45; 14.27)
**L/BMI**	1.5 (2.1; 1.2)	0.85 (1.18; 0.74)
**Adiponectin (A)**	8.4 (9.7; 5.96)	10.1 (14.4; 8.8)
**Adiponectin/Leptin (A/L)**	0.14 (0.25; 0.1)	0.6 (0.8; 0.4)
**Caro index (mmol/l/μEd/ml)**	0.3 (0.39; 0.27)	0.62 (0.75; 0.51)
**HOMA-IR**	2.1 (4.6; 1.5)	1.56 (1.7; 1.4)
**HOMA-AD**	6.7 (9.3; 3.4)	2.8 (3.1; 2.5)

In group I, the level of adiponectin exceeded the values of the control group (p<0.05), the indicators of which did not differ statistically (p>0.05) (Table 1). It is known that the gene responsible for the synthesis of adiponectin is located on chromosome 3q27 at the locus associated with visceral obesity and metabolic syndrome, which fully explains the relationship between low values of adiponectin with elevated MI in patients from group I, confirmed by the results of correlation analysis (adiponectin & BMI: ρ=-0.74).

It is important to find prognostic markers of ovarian dysfunction formation. Currently, there is an opinion about the need to develop a more accurate indicator of a quantitative assessment of IR than the HOMA-IR index, which is considered insufficiently informative according to some authors. In the works of Zaletel *et al.* [13], there are indications of the possibility of indirect determination of IR and severity of metabolic syndrome by the ratio of adiponectin/leptin (A/L), which is a more effective indicator of IP than a single determination of adiponectin, leptin or HOMA-IR in patients without diabetes. The analysis of the obtained results revealed a decrease in A/L in group I – 5.4 times compared with patients in group II (p<0.05) and 4.3 times – compared with the control group (p<0.05). In group II and the control group, this indicator was not statistically justified (p<0,05) (Table 1). In 2007, Matsuhisa *et al.*, based on the role of adiponectin in the pathogenesis of IP, proposed to modify the HOMA-IR index in HOMA-AD (fasting glucose (mmol/l) x IRI on an empty stomach (μED/ml)/adiponectin) [14]. The results revealed a decrease in A/L in group I – 4.3 times – compared with girls in the control group (p<0,05). We calculated HOMA-AD in all clinical groups (Table 1) and found an increase in the main group compared with the control group 2.4 times (p<0, 05). With the A/L and HOMA-AD models, the selection of patients for this type of treatment will be more thorough, which will help avoid polypragmatism and improve treatment tactics.

We found that in adolescents with a regular menstrual cycle, normal BMI, and normal insulin sensitivity, the ratio of A/L>0.3. With MFI, hyperandrogenemia, obesity, and IR in adolescents, the value of this indicator is less than the established diagnostic threshold – the critical level – ≤0.3. If the values of A/L>0.3, treatment tactics may be limited to diet therapy and exercise. In addition to diet and exercise, the treatment complex should include drug therapy – insulin sensitizers at a lower level. The accuracy of the decision tree means the ratio of correctly classified objects to their total number, and the error – the number of incorrectly classified objects. The diagnostic accuracy of this classification model is 94.2%, sensitivity – 93.3%, specificity – 94.4%. Analyzing the indicators of A/L in the main group, we found that the values of this indicator in 94% of patients were below the established diagnostic threshold, which indicates the high diagnostic accuracy of this method.

We found that with a regular menstrual cycle, the level of HOMA-AD corresponds to values<3.45 with no hyperandrogenism and IR. When HOMAAD values are below this threshold – the prognosis of menstrual recovery is favorable, treatment aimed at normalizing insulin sensitivity is assessed as effective. At levels ≥3.45, insulin sensitizers should be included in the patient’s treatment. The diagnostic accuracy of this classification model is 94.2%, sensitivity – 93.3%, specificity – 94.4%. In retrospect, we found that in 94% of patients in the main group, this figure exceeded the established diagnostic threshold, which indicated the presence of IR.

### Rationale dividing main group in 2 groups: I and II

As a result of these objective and clinical symptoms among patients in the main group (79 obese girls), there are two groups: I with apparent signs of hyperandrogenism (46 patients) and II (33) – without clinical manifestations of hyperandrogenism. Clinical manifestations of hyperandrogenism in groups I and II. The results obtained are shown in Table 2.

**Table 2.** Data on the hormonal status of patients in groups I and II (M±m) function associated with obesity.

**Index**	**Group I (n=46)**	**Group II (n=33)**	**Laboratory reference values**
**LH, MO/l**	18.1±0.22 *	8.0±0.51	1.9–12.5
**FSH, MO/l**	6.3±0.16	5.7±0.7	2.5–10.2
**LH/FSH**	2.8±0.6	1.7±0.8	≥2.5
**Prolactin, μMO/ml**	242.9±11.14 *	421.1±18.6	59–619
**Testosterone, nmol/l**	4.9±0.19 *	2.2±0.7	0.5–2.6
**Dehydroepiandrosterone disulfate, μmol/l**	8.2±0.9	5.1±0.08	1.0–11.7
**TSH, μMO/ml**	1.4±0.08	2.1±8.7	1.0–3.5
**Estradiol, pmol/l**	229.5±12.09	196.3±16.8	150–480
**Insulin (fasting) μEU/ml**	14.1±0.08 *	6.2±0.07	2.7–10.4

* – significance of differences between 1 and 2 groups (p1-2 <0.05).

In group I, the testosterone level was higher than normal by 2.3 nmol/l, 88.4% of the maximum limit of normal, and significantly (p<0.05) exceeded the same indicator in group II by almost two times. The study of dehydroepiandrostenedione sulfate revealed that it was slightly higher than in the group without hyperandrogenism but remained within the reference values, which indicated the predominance of the ovarian genesis of hyperandrogenic symptoms in group I. In addition, the analysis of the ratio of LH/FSH revealed that this figure was significantly higher in the group of patients with hyperandrogenism than in group II. Although many scientists have recently denied this diagnostic sign of polycystic ovary syndrome (PCOS), this fact may indicate in favor of developing polycystic ovaries in women with hyperandrogenism from group I. This was confirmed by the data of ultrasound examination of morphology, size, and volume of the ovaries in groups. Noteworthy is the slightly increased prolactin content, significantly higher in the blood plasma of group II but without exceeding the norm (Table 2). This appears to be due to hormone levels exceeding reference values, observed in 9 of 32 (28.1%) patients and corresponded to moderate hyperprolactinemia. Due to the lack of data on pituitary damage and a slight increase in prolactin concentration, this symptom was interpreted as transient hyperprolactinemia in these patients. Given that one of the mechanisms of PCOS pathogenesis is insulin resistance, we studied our patients’ fasting insulin levels, blood glucose levels of patients in both groups, and the HOMA index (Table 3).

**Table 3.** Blood glucose levels of patients in both groups and the HOMA index.

**Index**	**Group I (n=46)**	**Group II (n=33)**	**Pearson – criterion**
**Blood glucose level**	35 (76%)	31 (96.8%)	0.0129 *
**Impaired glucose tolerance**	11 (23.9%)	1 (3.12%)	0.0412 *
**Hyperinsulinemia**	12 (26 %)	0	0.0513 *
**HOMA index is normal (norm 3.3–3.8)**	34 (73.9%)	31 (96.8%)	0.8479

* – statistically significant difference (p<0.05); _χ_2, Pearson Pearson’s _χ_2 criterion.

Given the group’s heterogeneity in the severity of obesity, patients in groups I and II were divided by the degree of obesity using BMI to diagnose metabolic disorders and subsequently assess the effectiveness of treatment. Differences in leptin levels between the groups and depending on the degree of obesity were reliably detected (Table 4).

**Table 4.** The difference in leptin level between the groups.

BMI	Leptin (ng/ml) Group A1	Leptin (ng/ml) Group A2
**Norm (1.1–27.6 ng/ml)**		
**Class I obesity**	32.36±1.1	35.8±2.4
**Class II obesity**	35.06±1.13	39.23±3.1
**Class III obesity**	38.14±1.8	43.14±2.2

Leptin levels increase according to the severity of obesity in both groups. However, it is significantly higher in all obesity classes in group II, reaching a concentration of 43.14±2.2 in obesity III.

## Discussion

It is well-known that menstrual function depends on the state of gonadotropic function of the pituitary gland (GtFG). The influence of dysgonadotropinemia (multidirectional changes in the levels of luteinizing and follicle-stimulating hormones (LH and FSH) on the formation of MFI in adolescents began in the early 2000s [15], and in recent years its role in the development of endocrine infertility in women has been established [16, 17].

The results obtained are consistent with the studies of Fasshauer [18] and Schwartz [19], who found hypoadiponectinemia in overweight people. Furthermore, in a few clinical studies, a correlation was found between serum adiponectin levels and the degree of IR, which is also confirmed by the results of our work (adiponectin & Caro index: ρ=-0.83 – in the main group of patients).

The discovery of a hormone such as leptin introduced a possible mechanism through which metabolic signals about nutritional status and the percentage of the fat component can be transmitted to the reproductive axis. Leptin is thought to be a “mediator of puberty”. Synthesis and secretion of GnRH, the formation of gonadotropin sensitivity to GnRH and the release of LH and FSH, steroidogenesis, proliferation of cell elements of the endometrium, MH, as well as ensuring programmed cell death, a list of processes dependent on leptin levels. It is a proven fact that restriction in eating behavior leads to a decrease in leptin level in the blood plasma of such patients, as normoleptinemia is a prerequisite for balanced activity of the entire reproductive system [20–23].

Thus, hyperleptinemia was found in patients with menstrual dysfunction associated with obesity but was more pronounced in patients without hyperandrogenism and class III obesity. This may have become the main pathogenetic mechanism of obesity in group II. Analyzing the obtained clinical and laboratory data, adolescent girls with obesity showed apparent differences in the severity of hyperandrogenic disorders of carbohydrate metabolism and insulin resistance.

Based on our results and studies by M. Matsuhisa [14] and A.M. Hung [24], A/L and HOMA-AD models can be considered more accurate for determining IR than HOMA-IR and Caro index. This conclusion is very significant because for the treatment of patients with IMF associated with obesity and detected IR (based on HOMA-IR models and Caro index), insulin sensitizers are often used, including metformin, the indications of which in children and adolescents are limited.

Undoubtedly, the first and most important stage of treatment for these patients is weight loss; non-drug and drug therapy was used for this purpose. The treatment was performed in 2 stages. Based on the pathogenesis, at stage 1, therapeutic measures were primarily aimed at weight loss. It is necessary to optimize reproductive function in obese women [25–28], which might positively affect general health. To restore regular menstrual function and ovulation in most cases, it is enough to reduce body weight by 10–15% from baseline or reduce BMI by 2–5 kg/m^2^ from the initial parameters. The recommendations for the patients are both standard low concentrated sweets diet and daily exercise (walking, swimming etc). Moreover, a low-calorie diet and increased physical activity are recommended. All women received recommendations on proper nutrition and motivation to change their eating behavior, lifestyle, and long-term support of the achieved result [29, 30].

## Conclusion

The association of adipokines secretion with obesity is characterized by hyperleptinemia, leptin resistance, decreased adiponectin/leptin index, and hypoadiponectinemia, combined with hyperandrogenism and insulin resistance indicates the participation of these adipokines in genesis oligomenorrhea. The development of menstrual disorders associated with obesity is based on endocrine disorders. The characteristic feature is dysgonadotropinemia manifested by high LH concentrations (78.3%), and its low levels in one of every five patients combined with normal or low levels of FSH. This indicates various mechanisms of disease development and may be a consequence of the ovarian or the hypothalamic-pituitary axis insufficiency.

## Acknowledgments

### Conflict of interest

The authors declare that there is no conflict of interest.

### Ethical approval

The approval for this study was obtained from the Ethics Committee of the HSEEU Bukovinian State Medical University and Yuzko Medical Center, Ukraine (approval ID: 12-06.09.2021). Our study was conducted according to the Declaration of Helsinki adopted in 1975 and revised in 2008, and the ethical principles were entirely respected.

### Consent to participate

Written informed consent was obtained from the participants in the study.

### Data availability

The data of this study is available by request.

### Authorship

AB was responsible for the clinical management of the patients. OB contributed to the methodology. AA contributed to the data analysis and drafting of the manuscript. VA conducted the statistical analysis. MS contributed to conceptualizing. VV contributed to editing the manuscript. OA was the project leader and was responsible for finalizing the manuscript.
